# Special Issue “New Advances in Insulin—100 Years Since Its Discovery”

**DOI:** 10.3390/biomedicines13092207

**Published:** 2025-09-09

**Authors:** Tomislav Bulum

**Affiliations:** 1 Department of Diabetes and Endocrinology, Vuk Vrhovac University Clinic for Diabetes, Endocrinology and Metabolic Diseases, Merkur University Hospital, Dugi dol 4a, 10000 Zagreb, Croatia; tomobulum@gmail.com; Tel.: +385-12353887; Fax: +385-12331515; 2 School of Medicine, University of Zagreb, Šalata 3, 10000 Zagreb, Croatia

The discovery of insulin in 1921 represented a milestone in the treatment of diabetes. In 1923, the Nobel Prize for Medicine was awarded for its discovery, and it is considered one of the most valuable scientific events of the 20th century, affecting millions of people worldwide [1,2,3]. Before the discovery of insulin, children diagnosed with type 1 diabetes typically survived for less than a year after diagnosis, and those with type 2 diabetes also faced significantly reduced life expectancy. At that time, treatment options were extremely limited, and patients were often placed on strict diets with minimal carbohydrate intake in an attempt to control their symptoms. While this approach could slightly prolong patients’ lives, it was not a cure and, in some cases, it led to death by starvation due to severe caloric restriction.

Since insulin discovery, thousands of lives have been saved, and the life expectancy of people with diabetes has been significantly extended. Since its discovery, insulin has been continuously improved through pharmacological development and optimized for therapeutic purposes, including the development of intermediate- and long-acting insulins, the production of human insulin, and finally, the development of insulin analogs with improved properties using recombinant-DNA technology [4].

Despite over a century of using insulin as a standard treatment for type 1 diabetes—with continuing advances in diabetes research and technology, continuous glucose monitoring and insulin administration via insulin pumps—we are far from being able to achieve a real cure for type 1 diabetes. Moreover, in the last 15 years, the number of people diagnosed with type 1 diabetes has increased by 45% [5,6]. Despite the rapid development of a wide range of new drugs for the treatment of diabetes, particularly in the context of protection from cardiorenovascular diseases, insulin remains one of the most potent therapeutic options for patients with type 2 diabetes, and the only treatment for those with type 1 diabetes [7]. In addition, despite increasing awareness of diabetes and its devastating complications, it is one of the leading causes of death and disability worldwide, irrespective of gender, age, and country, and health expenditures associated with diabetes and its chronic complications will reach more than USD 1054 billion by 2045 [8].

Although recent trends show a decline in complications among patients with type 1 diabetes—largely due to improved management practices and advancements in care technologies such as continuous glucose monitoring and modern insulin delivery systems—the risk of microvascular and macrovascular complications remains high [9,10]. In this context, this Special Issue aims to highlight the latest research and advances related to insulin and its actions in a broad sense, featuring six original research articles, four review articles, and one systematic review.

The first research article investigated potential factors associated with post-bariatric hypoglycemia. It is well known that post-bariatric hypoglycemia is a challenging condition affecting the quality of life of patients after bariatric surgery [11]. In this study, risk of post-bariatric postprandial hypoglycemia was observed over 12 months in a cohort of 24 patients with type 2 diabetes mellitus and a body mass index of ≥40 kg/m^2^ who had undergone laparoscopic Roux-en-Y gastric bypass. All patients filled in a questionnaire based on the Edinburgh hypoglycemia scale. The results of the study suggest that pre-existing β-cell hyperfunction, which persists postoperatively after weight loss, and consequent postprandial insulin values at 30 min and 6 months seem to be strong predictors for post-bariatric hypoglycemia, while GLP-1 and glucagon values were not significantly associated with post-bariatric hypoglycemia.

The second research article was a real-world, retrospective study of patients with type 1 diabetes using multiple daily insulin injections. The study aimed to assess overnight glucose levels based on insulin type and timing. Nocturnal hypoglycemia is frequent, and episodes are often prolonged for several hours, particularly in those who undertake physical activity during the day [12]. In this study, continuous glucose monitoring and insulin injection data were collected for ten hours after dinner using the Insulclock^®^ connected cap. Higher glucose was observed in subjects with delayed injections, while those using ultrarapid insulin had fewer hypoglycemic events. Those on glargine U300 had a higher time in range than those on degludec, while use of a correction injection was associated with a higher number of hypoglycemic events. The results of the study suggest that the time of rapid insulin injection before dinner, insulin type, and use of correction injections affect nocturnal glucose profiles in patients with type 1 diabetes.

The third research article was a pilot study that investigated the relationship between the proteomics profiles of serum exosomes from normal individuals and those with obesity and insulin resistance. The researchers identified 23 upregulated and 46 downregulated proteins between normal individuals and those with obesity and insulin resistance. Those affecting insulin resistance, β-cell activation, and inflammation were upregulated in subjects with obesity and insulin resistance compared to lean/overweight insulin-sensitive subjects. The researchers concluded that in subjects with obesity and insulin resistance, serum exosomal proteins can be used as biomarkers to identify the future risk of diabetes and as a therapeutic target to prevent or slow down the progression of diabetes.

The fourth research article explores the effect of insulin and metformin on the mortality of patients with type 2 diabetes with symptomatic COVID-19 infection. Previous observational analyses have shown that prevalent metformin use is associated with favorable outcomes after COVID-19 infection in adults with type 2 diabetes [13]. On the other hand, insulin treatment is associated with increased mortality in patients with COVID-19 and type 2 diabetes [14]. In this study, the association of death with insulin and metformin therapy was weak and could not be included in the multivariate model. However, the interaction of both drugs with other factors, including remdesivir and low-molecular-weight heparin (metformin), age, and C-reactive protein (insulin), modulated the odds of death. The role of metformin and insulin in the mortality of patients with type 2 diabetes and symptomatic COVID-19 may vary depending on a more personalized risk assessment model that includes and explores their interactions with various patient characteristics. In contrast, most other studies examining metformin and insulin in the context of COVID-19-related mortality lack such an exploration of interaction effects.

The fifth research article investigated the risk factors for lower transcutaneous oxygen pressure as a measure of microvascular circulation in the feet of patients with type 2 diabetes. Transcutaneous oxygen pressure is an excellent prognostic tool for wound healing in the population of diabetic patients with foot problems [15]. This study included 119 patients with type 2 diabetes. Those with lower transcutaneous oxygen pressure (<40 mmHg), indicating lower tissue oxygenation and a risk of delayed wound healing, were younger; had higher systolic blood pressure, glycated hemoglobin, fasting plasma glucose, and total and LDL cholesterol; and smoked more frequently than those with normal transcutaneous oxygen pressure. However, in association with other risk factors, smoking was the main predictor for lower transcutaneous oxygen pressure in patients with type 2 diabetes. Additional efforts are needed in everyday clinical practice to actively encourage patients with type 2 diabetes and peripheral arterial disease to quit smoking.

The sixth research article examined the relationship between hyperglycemia and prognostic value in patients with COVID-19 infection admitted to a hospital in Lithuania. Admission hyperglycemia is a predictor of mortality in patients hospitalized with COVID-19, even in patients without a previous history of diabetes, and hyperglycemia is also associated with increased mortality in critically ill patients with COVID-19 [16,17]. This study aimed to evaluate the association between hyperglycemia at admission with the need for invasive mechanical ventilation and in-hospital mortality in patients without diabetes who were hospitalized for COVID-19 infection. Over 10% of hospitalized patients had intermittent hyperglycemia at admission (blood glucose levels ≥ 7.8 mmol/L and <11.1 mmol/L), and those patients had a hazard ratio of 3 for 30-day in-hospital mortality compared to those with normoglycemia at admission.

The first narrative review comprises data from preclinical animal studies, longitudinal cohort studies, cross-sectional studies, machine learning analyses, and randomized controlled trials. It examines the role of insulin in maintaining health and preventing disease. This review proposes an expanded view of insulin as a metabolic–socio-psychological substance within the mitochondrial information processing framework, suggesting its roles beyond metabolism, particularly in cooperating with mitochondria to process psychosocial factors into the biological fabric, and providing new insights into the interplay between socio-psychological factors and biological systems in chronic diseases, such as type 2 diabetes.

The second review examines the potential impact of insulin, metformin, and glucagon-like peptide-1-based therapies on the progression of osteoarthritis in patients with diabetes. Evidence suggests that diabetes and osteoarthritis coexist within the same population, and that diabetes is associated with a greater degree of osteoarthritic pain [18]. The results of this review indicate that insulin, beyond its role in glycemic control, may potentially influence joint health by modulating inflammatory pathways relevant to osteoarthritis. Metformin also shows promise in mitigating osteoarthritis via its anti-inflammatory properties and reducing inflammatory markers. Glucagon-like peptide-1-based therapies also exhibit anti-inflammatory effects that may suppress cytokine-mediated joint inflammation and support cartilage repair mechanisms, with beneficial effects on osteoarthritis. Targeting common underlying mechanisms, insulin, metformin, and glucagon-like peptide-1-based therapies have therapeutic potential in osteoarthritis.

The third review focuses on the role of basal weekly insulin in clinical practice, and its use in patients with type 1 and 2 diabetes, by examining its safety, efficacy, and manageability, and therapeutic compliance. Several new weekly insulins have been developed, and they have demonstrated a significant decrease in blood glucose in pre-clinical studies. Therapy with basal weekly long-acting insulin in patients with type 1 and type 2 diabetes shows similar and better glycemic efficacy than daily basal insulin due to its association with reduced hypoglycemia, a reduction in the number of injections, and its proven effectiveness. Icodec insulin would likely be the most effective primary basal weekly insulin, as supported by the results of the ONWARDS clinical program, due to its tolerability, safety in terms of hypoglycemia, and increased patient compliance [19].

The fourth review summarized the last 100 years of insulin research, from its discovery to the insulins of the future. The discovery of insulin had a profound impact not only within the field of diabetes but also across medicine as a whole, emphasizing the importance of collaboration between clinicians, researchers, and pharmaceutical companies committed to improving the lives of people with diabetes. Notably, decision of the original discover not to commercialize or profit from their discovery enabled rapid and widespread progress in the early years. This stands in contrast with more recent developers who have patented new insulin formulations, potentially limiting their broader accessibility. Although a century has passed since the discovery of insulin and significant progress has been made in diabetes research, a true cure for type 1 diabetes—one that prevents the autoimmune destruction of pancreatic beta cells—remains elusive. Nevertheless, ongoing advancements continue to improve the quality of life for individuals living with the disease year after year.

The final review is a systematic review that explores the efficacy and safety of Icodec compared to once-daily insulin analogs (Degludec U100, Glargine U100, Glargine U300, and Detemir) in type 1 and type 2 diabetes. The review included 4347 patients with type 1 and type 2 diabetes with glycated hemoglobin over 7%. Those treated with Icodec had a greater probability of achieving glycated hemoglobin A1c < 7% without severe hypoglycemic events, but with slight and statistically significant weight gain. No difference in fasting glucose levels, time in range, and time above range was observed. Icodec demonstrated slightly greater efficacy compared to its competitors when used in a basal-only regimen rather than in a basal-bolus approach. While weight gain and the risk of hypoglycemia were relatively low, they remain clinically relevant and should not be overlooked.

In conclusion, this Special Issue offers a comprehensive overview of insulin secretion and action, as well as the development and action of insulin analogs and their impact on glycemic control and the chronic complications of diabetes. Advances in diabetes treatment have been numerous in the 100 years since the discovery of insulin, resulting in extraordinary progress in the development of novel molecules to improve glucose control, simplify insulin regimens, and enhance quality of life. However, insulin remains the only replacement therapy for type 1 diabetes, and the data presented here may encourage and support further research in this important field.
